# AI-Based Early Change Detection in Smart Living Environments

**DOI:** 10.3390/s19163549

**Published:** 2019-08-14

**Authors:** Giovanni Diraco, Alessandro Leone, Pietro Siciliano

**Affiliations:** CNR—National Research Council of Italy, IMM—Institute for Microelectronics and Microsystems, 73100 Lecce, Italy

**Keywords:** artificial intelligence, machine learning, deep learning, smart living, multi-sensor system, big data analytics, change detection, human behavior, activity of daily living, ambient assisted living

## Abstract

In the smart environments we live today, a great variety of heterogeneous sensors are being increasingly deployed with the aim of providing more and more value-added services. This huge availability of sensor data, together with emerging Artificial Intelligence (AI) methods for Big Data analytics, can yield a wide array of actionable insights to help older adults continue to live independently with minimal support of caregivers. In this regard, there is a growing demand for technological solutions able to monitor human activities and vital signs in order to early detect abnormal conditions, avoiding the caregivers’ daily check of the care recipient. The aim of this study is to compare state-of-the-art machine and deep learning techniques suitable for detecting early changes in human behavior. At this purpose, specific synthetic data are generated, including activities of daily living, home locations in which such activities take place, and vital signs. The achieved results demonstrate the superiority of unsupervised deep-learning techniques over traditional supervised/semi-supervised ones in terms of detection accuracy and lead-time of prediction.

## 1. Introduction

Frail subjects, such as elderly or disabled people, may be at risk when their health conditions are amenable to change, as it is quite common in case of chronic conditions. That risk can be reduced by early detecting changes in behavioral and/or physical state, through sensing and assisted living technologies, nowadays widely available in smart-living environments. Such technologies, indeed, are able to collect huge amounts of data by days, months, and even years, yielding meaningful information useful for early detection of changes. Furthermore, early detection makes it possible to alert relatives, caregivers, or health-care personnel in advance when significant changes or anomalies are detected, and above all before critical levels are reached. The “big” data collected from smart homes offer a significant opportunity to assist people for early recognition of symptoms that might cause more serious disorders, and so preventing chronic diseases. The huge amounts of data collected by different devices require automated analysis, and thus it is of great interest to investigate and develop automatic systems for detecting abnormal activities and behaviors in the context of elderly monitoring [1] and smart living [2] applications.

Moreover, long-term health monitoring and assessment can benefit from knowledge held in long-term time series of daily activities and behaviors as well as physiological parameters (PPs) [3]. From a big data perspective, the main challenge is to process and automatically interpret—obtaining quality information—the data generated, at high velocity (i.e., high sample rate) and volume (i.e., long-term datasets), by a great variety of devices and sensors (i.e., structural heterogeneity of datasets), becoming more common with the rapid advance of both wearable and ambient sensing technologies [4]. A lot of research has been done in the general area of human behavior understanding, and more specifically in the area of daily activity/behavior recognition and classification as normal or abnormal [5,6]. However, very little work is reported in the literature regarding the evaluation of machine learning (ML) techniques suitable for data analytics in the context of long-term elderly monitoring in smart living environments. The purpose of this paper is to conduct a preliminary study of the most representative machine/deep learning techniques, by comparing them in detecting abnormal behaviors (ABs) and change prediction (CP).

The rest of this paper is organized as follows. The following Section 2 contains related works, some background and state-of-the-art in abnormal activity and behavior detection and CP, with special attention paid to elderly monitoring through big data collection and analysis. Section 3 describes materials and methods that have been used in this study, providing an overview of the system architecture, long-term data generation, and compares ML techniques. The findings and related discussion are presented in Section 4 and discussed in Section 5. Finally, Section 6 draws some conclusions and final remarks.

## 2. State-of-the-Art and Related Work

**Figure 1:**
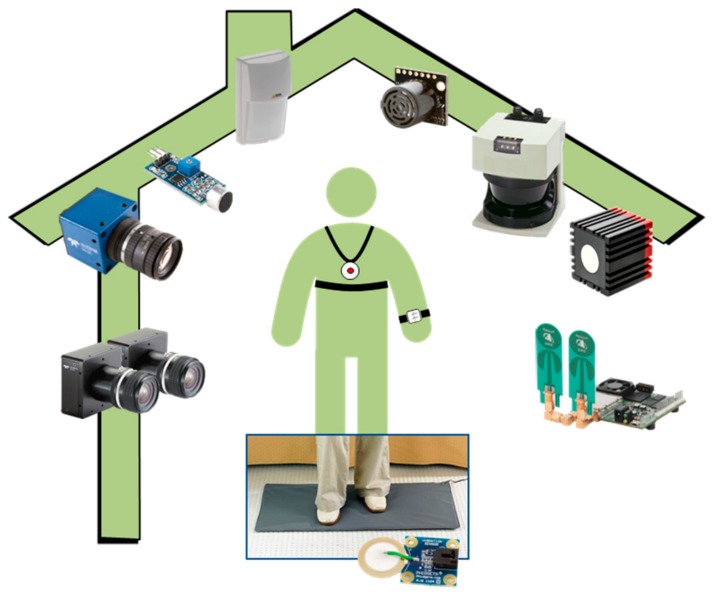
In health-smart homes, ambient and wearable technologies can produce a huge amount of data. From the left-hand side, the sensors are stereo-cameras, a monocular camera, microphone, pyroelectric infrared (PIR), sonar, lidar, time-of-flight camera, ultra-wideband radar, and a pressure/vibration sensor (on the floor).

Today’s available sensing technologies enable long-term continuous monitoring of activities of daily living (ADLs) and PPs (e.g., heart rate, breathing, etc.) in the home environment. As shown in Figure 1, both wearable and ambient sensing can be used, either alone or combined, to form multi-sensor systems [7]. In practice, wearable motion detectors incorporate low-cost accelerometers, gyroscopes, and compasses, whereas detectors of PPs are based on some kind of skin-contact biosensors (e.g., heart and respiration rates, blood pressure, electrocardiography, etc.) [8]. These sensors need to be attached to a wireless wearable node, carried or worn by the user, needed to process raw data and to communicate detected events with a central base station. Although wearable devices have the advantage of being usable “on the move” and their detection performance is generally good (i.e., signal-to-noise ratio sufficiently high), their usage is nonetheless limited by battery life time (shortened by the intensive use of the wireless communication and on-board processing, both high energy-demanding tasks) [9], by the inconvenience of having to remember to wear a device, and by the discomfort of the device itself [10].

Ambient sensing devices, on the other hand, are not intrusive in terms of body obstruction, since they require the installation of sensors around the home environment. Such solutions disappear into the environment, and so are generally well-accepted by end-users [10]. However, the detection performance depends on the number and careful positioning of ambient sensors, whose installation may require modification or redesign of the entire environment. Commonly used ambient sensors are simple switches, pressure and vibration sensors, embedded into carpets and flooring, particularly useful for detecting abnormal activities like falls, since elderly people are directly in contact with the floor surface during the execution of ADLs [11]. Ultra-wideband (UWB) radar is a novel, promising, unobtrusive and privacy-preserving ambient-sensing technology that allows to overcome the limitations of vision-based sensing (e.g., visual occlusions, privacy loss, etc.) [12], enabling remote detection (also in through-wall scenarios) of body movements (e.g., in fall detection) [13], PPs [14], or even both simultaneously [15].

As mentioned so far, a multi-sensor system for smart-home elderly monitoring needs to cope with complex and heterogeneous sources of information offered by big data at different levels of abstraction. For this purpose, in order to detect behavioral changes and abnormalities using a multi-sensor system, it is more appropriate to have an algorithmic framework able to deal with heterogeneous sensors by means of a suitable abstraction layer [16], instead having to design a data fusion layer developed for specific sensors [17].

The algorithmic techniques for detecting ABs and related changes can be roughly categorized into three main categories: Supervised, semi-supervised, and unsupervised approaches. In the supervised case, abnormalities are detected by using a binary classifier in which both normal and abnormal behavioral cues (e.g., sequences of activities) are labelled and used for training [18,19,20]. The problem with this approach is that ABs are extremely rare in practice, and so they must be simulated or synthetically generated in order to train models. In the semi-supervised case, only one kind of label is used to train a one-class classifier [21,22]. The advantage here is that only normal behavioral cues, which can be observed during the execution of common ADLs, are enough for training. The last but not least important category includes the unsupervised classifiers, whose training phase does not need labelled data at all (i.e., neither normal nor abnormal cues) [23]. In this case, the pro is that unsupervised detection can easily adapt to various environmental and user’s conditions, but the con that it requires a large amount of initial observations to be fully operational when operating for the first time [24,25].

In [26] the main supervised, semi-supervised, and unsupervised approaches for anomaly detection were investigated, comparing both traditional ML and deep learning (DL) techniques. The authors demonstrated the superiority of unsupervised approaches, in general, and of DL ones in particular. However, since that preliminary study considered simple synthetic datasets, further investigations are required to accurately evaluate the performance of the most promising traditional and DL methods under larger datasets, i.e., big data in long-term monitoring, including more variability in the data.

## 3. Materials and Methods

The present investigation is an extension of the preliminary study [26] that compared traditional ML and DL techniques on both abnormality detections and CPs. For each category of learning approach, i.e., supervised, semi-supervised, and unsupervised, one ML-based and one DL-based technique were evaluated and compared in terms of detection accuracy and prediction lead-time at the varying of both normal ADLs (N-ADLs) and abnormal ADLs (A-ADLs). All investigated ML–DL techniques are summarized in Table 1. For that purpose, a synthetic dataset was generated by referring to common ADLs and taking into account how older people perform such activities at their home environment, following instructions and suggestions provided by consultant geriatricians and existing researches [19,27]. The synthetic dataset included six basic ADLs, four home locations in which these activities usually take place, and five levels of basic PPs associated with the execution of each ADL.

As an extension of a previous study [26], the objective of this investigation is to evaluate more deeply the techniques reported in Table 1 by considering six additional abnormal datasets, instead of only one, obtained in the presence of the following changes:

- [St] Starting time of activity. This is a change in the starting time of an activity, e.g., having breakfast at 9 AM instead of 7 AM as usual.
- [Du] Duration of activity. This change refers to the duration of an activity, e.g., resting for 3 hours in the afternoon, instead of 1 hour as usual.
- [Di] Disappearing of activity. In this case, after the change, one activity is no more performed by the user, e.g., having physical exercises in the afternoon.
- [Sw] Swap of two activities. After the change, two activities are per-formed in reverse order, e.g., resting and then housekeeping instead of housekeeping and resting.
- [Lo] Location of activity. One activity usually performed in a home location (e.g., having breakfast in the kitchen), after the change is performed in a different location (e.g., having breakfast in bed).
- [Hr] Heartrate during activity. This is a change in heartrate during an activity, e.g., changing from a low to a high heartrate during the resting activity in the afternoon.

Without loss of generality, generated datasets included as a PP only the heartrate (HR), since heart and respiration rates are both associated with the performed activity. The discrete values assumed by ADLs, locations, and heartrate values included in the generated datasets are reported in Table 2. It is important to note that the presence of these kinds of changes, in general, cannot be considered in itself as an abnormal status. Nonetheless, a sustained change over days or months in activities, locations, and vital signs may be linked to an AB. Within the application domain of ambient assisted living, the aim of this study is to evaluate the ability of various ML/DL methods in predicting such sustained changes, with the objective to notify caregivers/doctors who can use historical sensor data to make decisions.

Furthermore, in this study, both normal and abnormal long-term datasets (i.e., lasting one year each) are realistically generated by suggesting a new probabilistic model based on the Hidden Markov Model (HMM) and Gaussian process (GP). Finally, the evaluation metrics used in this study include, besides the accuracy (the only one considered in the previous study [26]), also the precision, sensitivity, specificity, and F1-score:

(1) $$\mathrm{accuracy}=\frac{\mathrm{TP}+\mathrm{TN}}{\mathrm{TP}+\mathrm{TN}+\mathrm{FP}+\mathrm{FN}},$$

(2) $$\mathrm{precision}=\frac{\mathrm{TP}}{\mathrm{TP}+\mathrm{FP}},$$

(3) $$\mathrm{sensitivity}=\frac{\mathrm{TP}}{\mathrm{TP}+\mathrm{FN}},$$

(4) $$\mathrm{specificity}=\frac{\mathrm{TN}}{\mathrm{TN}+\mathrm{FP}},$$

(5) $$F1\text{-}\mathrm{score}=2\times \frac{\mathrm{TP}}{2\times \mathrm{TP}+\mathrm{FP}+\mathrm{FN}},$$

where TP is the number of true positives, FP is the number of false positives, TN is the number of true negatives, and FN is the number of false negatives. Furthermore, the prediction performance has been evaluated in terms of “lead-time of prediction” defined as the maximum number of days, prior to the day on which the change becomes stable, within which the future change can be predicted with the highest performance scores (i.e., maximizing TP and TN, and minimizing FP and FN). Thus, the higher the lead-time (in days), the better the prediction performance.

In the following section, details concerning data generation, supervised, semi-supervised, and unsupervised ML/DL techniques for abnormal behavior detection (ABD) and CP are presented.

### 3.1. Data Generation

**Figure 2:**
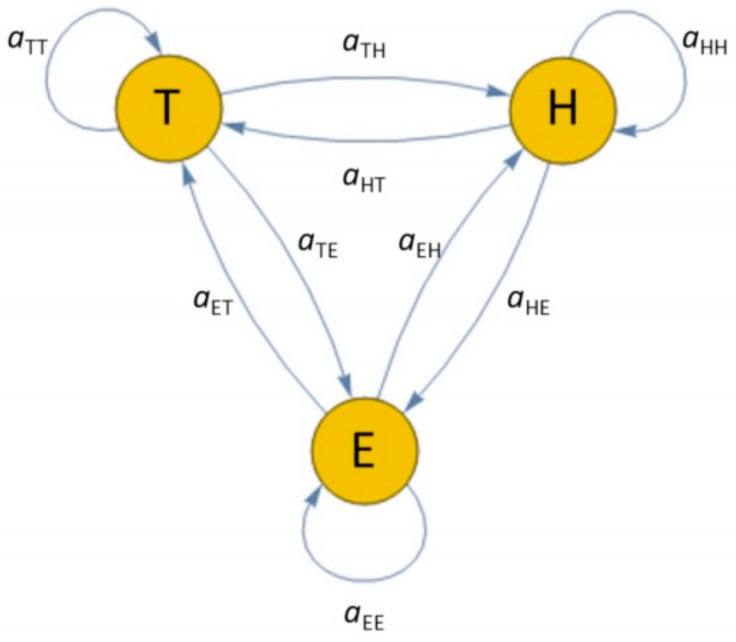
State diagram of the Hidden Markov Model (HMM) used to generate long-term activity data.

In the related literature, depending if the HMM is used for detecting ABs or activities, the hidden states are either behavioral statuses (e.g., normal, abnormal, critical) [19] or daily activities (e.g., eating, sleeping, etc.) [28]. Since, in this study, the HMM was used for data generation and not for detection purposes, the suggested model should be able to take into account the influence of circadian rhythms on motivated behaviors (e.g., sleep, hunger, exercise, etc.) [29]. For this purpose, the normal daily behavior has been modelled by using an HMM with three hidden states, Tired (T), Hungry (H), and Energized (E), as depicted in Figure 2, representing the user’s physical state bearing diverse ADLs during the daily circadian cycle. In such a way, each state can lead to different activities depending on user’s habits and the time of the day (e.g., the same state Tired can lead to the Sleeping activity in the night and to the Resting activity in the afternoon). Each arrow of the graph reported in Figure 2 is associated with a probability parameter, which determines the probability that one state $\pi_{i}$ follows another state $\pi_{i\;-\;1}$, i.e., the transition probability:

(6) $$a_{qr}=P\left(\pi_{i}=q|\pi_{i\;-\;1}=r\right),$$

where $q,r\in \left\{T,H,E\right\}$. The HMM output is a sequence of triples $\left(a,b,c\right)\in ADL\times LOC\times HRL$, where $ADL=\left\{AE,AH,AP,AR,AS,AT\right\}$, $LOC=\left\{BR,KI,LR,TO\right\}$, and $HRL=\left\{VL,LO,ME,HI,VI\right\}$ represent, respectively, all possible ADLs, home locations, and HR levels (see Table 2). In general, a state can produce a triple from a distribution over all possible triples. Hence, the probability that the triple (a, b, c) is seen when the system is in state k, i.e., the so-called emission probability, is defined as follows:

(7) $$e_{k}\left(a,b,c\right)=P\left(x_{i}=\left(a,b,c\right)|\pi_{i}=k\right).$$

**Figure 3:**
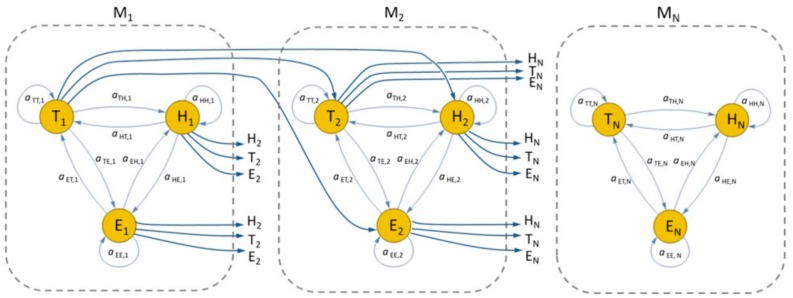
State diagram of the suggested hierarchical HMM, able to model the temporal dependency of daily activities.

Since HMM does not represent the temporal dependency of activity states, a hierarchical approach is proposed here by subdividing the day into N time intervals, and modeling the activities in each time interval with a dedicated HMM sub-model, namely $M_{1}$, $M_{2}$, …, $M_{N}$, as depicted in Figure 3. For each sub-model $M_{i}$, thus, the first state being activated starts at a time $T_{i}$ modeled as a GP, while the other states within the same sub-model $M_{i}$ start in consecutive time slots whose durations are also modeled as GPs.

**Figure 4:**
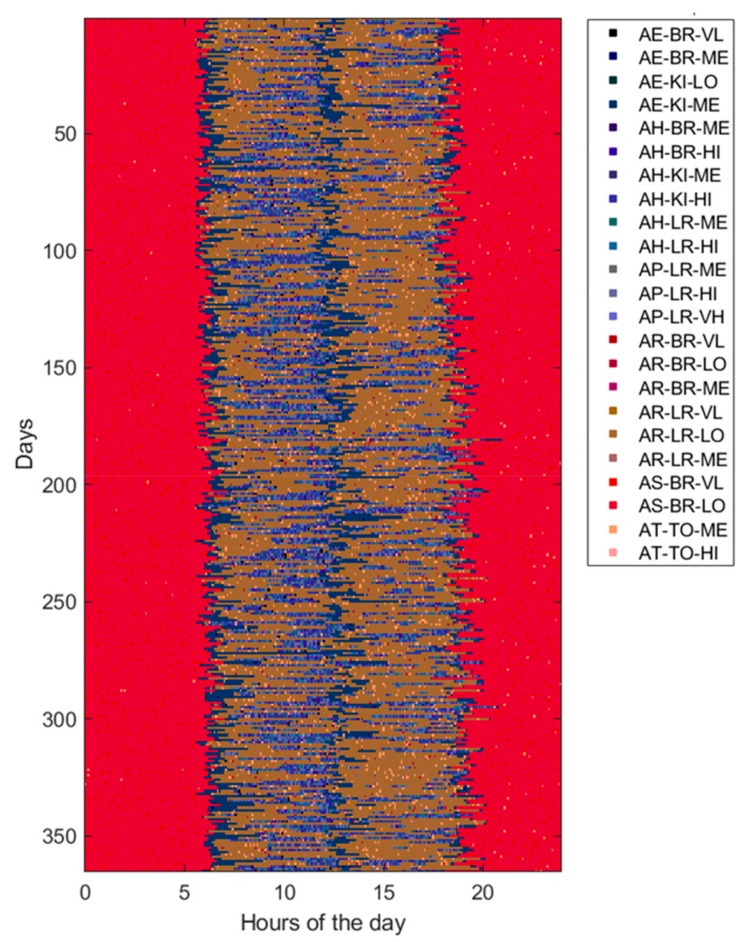
Example of a normal dataset represented as an image of 365 × 288 pixels and 120 levels (only used levels are reported in the legend).

**Figure 5:**
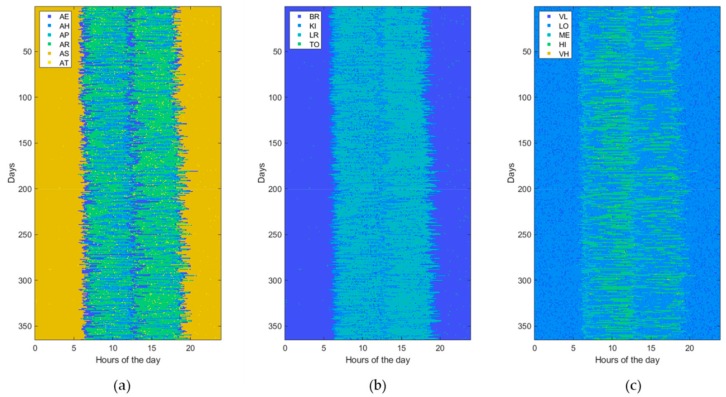
The same normal dataset shown in Figure 4 but represented with different images for (**a**) activity of daily living (ADL), (**b**) home locations (LOCs), and (**c**) heartrate levels (HRLs).

Usually, ADLs, home locations, and HR levels are sampled at different rates according to the specific variability during the entire day time. For example, since the minimum duration of the considered ADLs is of about 10 min, it does not make sense to take a sampling interval of 1 min for ADLs. However, for uniformity reasons, a unique sampling interval is adopted for all measurements. In this study, the HR sampling rate (i.e., one sample each 5 min) is selected as the reference to which the others are aligned by resampling them. Then, the generated data are prepared in a matrix form with rows and columns corresponding, respectively, to the total number of observed days (365 in this study) and to the total number of samples per day (288 in this study). Each matrix cell holds a numeric value that indicates a combination of values reported in Table 2, for example AE_KI_ME, indicating that the subject is eating her meal in the kitchen and her HR level is medium (i.e., between 80 and 95 beats/min). Thus, a 1-year dataset can be represented by an image of 365 × 288 pixels with 120 levels (i.e., 6 ADLs, 4 locations, and 5 HR levels), of which an example is shown in Figure 4. Alternatively, for a better understanding, a dataset can be represented by using three different images of 365 × 288 pixels, one for ADLs (with only 6 levels), one for locations (with only 4 levels), and one for HR levels (with only 5 levels), as shown in Figure 5.

Once the HMM has been defined, as discussed above, it was trained by using handcrafted label sequences (i.e., ADL, home location (LOC), and heartrate level (HRL) parameters), obtained following instructions and suggestions provided by consultant geriatricians and referring to existing researches [19,27]. Hence, the trained HMM was used to generate the synthetic datasets.

**Figure 6:**
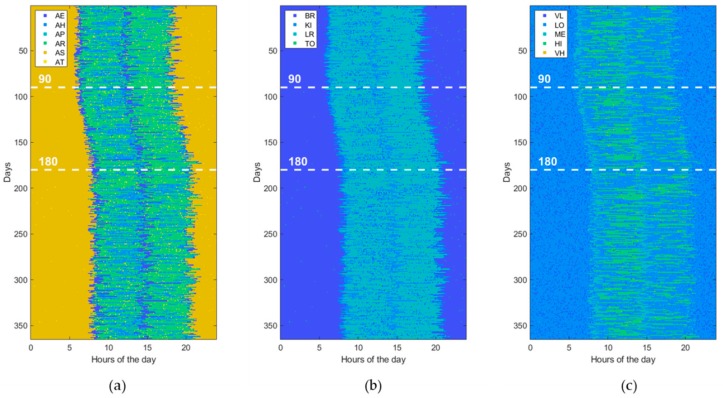
Example of an abnormal data set, due to a change in the “Starting time of activity” (St). The change gradually takes place from the 90th day on. (**a**) ADL, (**b**) LOCs, and (**c**) HRLs.

**Figure 7:**
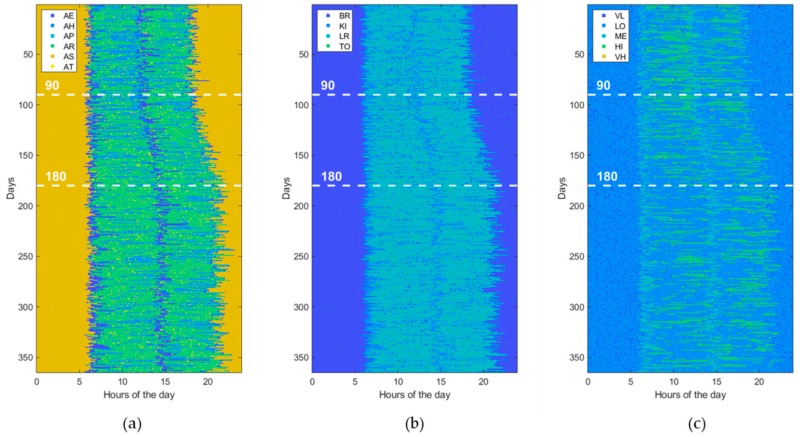
Example of an abnormal data set, due to a change in “Duration of activity” (Du). The change gradually takes place from the 90th day on. (**a**) ADL, (**b**) LOCs, and (**c**) HRLs.

**Figure 8:**
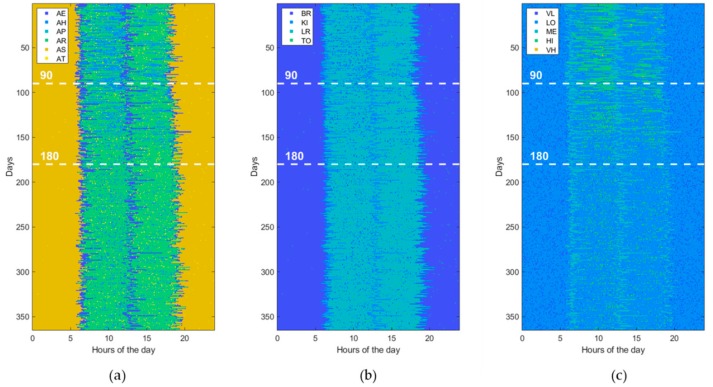
Example of an abnormal data set, due to a change in “Disappearing of activity” (Di). The change gradually takes place from the 90th day on. (**a**) ADL, (**b**) LOCs, and (**c**) HRLs.

**Figure 9:**
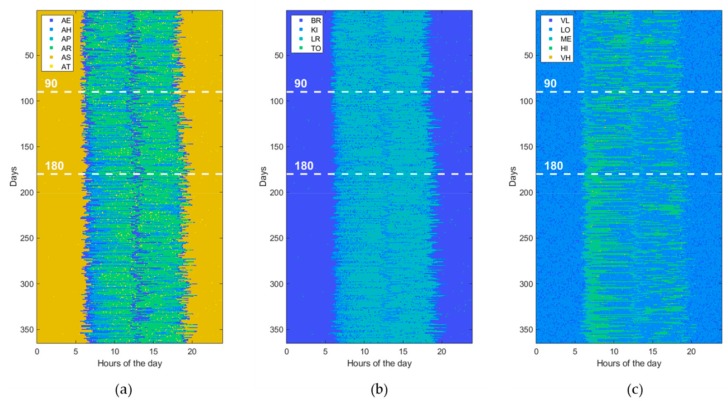
Example of an abnormal data set, due to a “Swap of two activities” (Sw). The change gradually takes place from the 90th day on. (**a**) ADL, (**b**) LOCs, and (**c**) HRLs.

**Figure 10:**
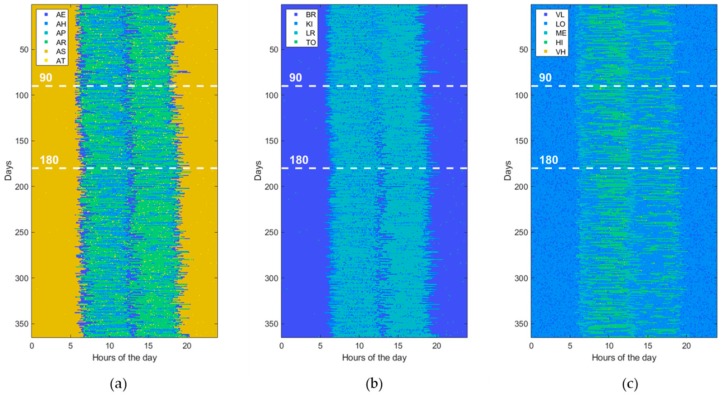
Example of an abnormal data set, due to a change in “Location of activity” (Lo). The change gradually takes place from the 90th day on. (**a**) ADL, (**b**) LOCs, and (**c**) HRLs.

**Figure 11:**
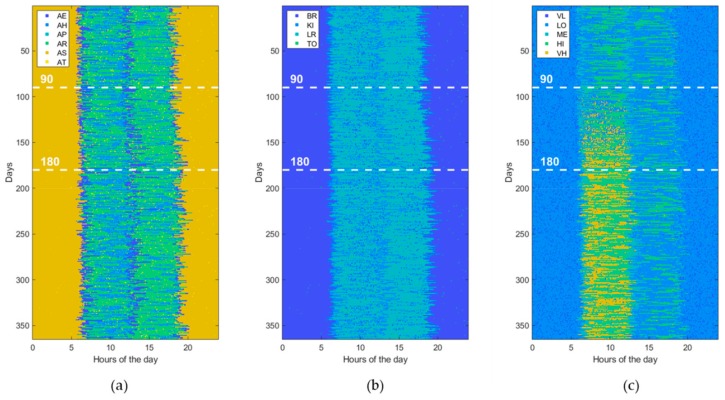
Example of an abnormal data set, due to a change in “Heartrate during activity” (Hr). The change gradually takes place from the 90th day on. (**a**) ADL, (**b**) LOCs, and (**c**) HRLs.

Furthermore, to assess the ability of ML and DL techniques (reported in Table 1) to detect behavioral abnormalities and changes, the model parameters (i.e., transition probabilities, emission probabilities, starting times, and durations) were randomly perturbed in order to generate various kind of abnormal datasets. Without loss of generality, each abnormal dataset included only one of the abovementioned changes (i.e., St, Du, Di, Sw, Lo, Hr) at a time or pairs of them, taken without repetitions (i.e., StDu, StDi, StSw, StLo, StHr, etc.). At this end, the perturbation is gradually applied between the 90th and 180th day, by randomly interpolating two sets of model parameters, normal and abnormal, respectively. Thus, an abnormal dataset consists of three parts. The first one, ranging from day 1 to day 90, is referred to normal behavior. The second period, from day 90 to 180, is characterized by gradual changes, becoming progressively more accentuated. Finally, the third period, starting from day 180, is very different from the initial normal period, the change rate is low or absent, and the subject’s behavior moves into another stability period. An example dataset for each abnormality (St, Du, Di, Sw, Lo, Hr) is reported in figures from Figure 6, Figure 7, Figure 8, Figure 9, Figure 10 and Figure 11. The detection performance of each technique is evaluated for different A-ADL levels (i.e., percentages of abnormal activities present in a dataset) as well as different prediction lead-time, which is the maximum number of days in advance such that the abnormality can be detected with a certain accuracy. Furthermore, in order to better appreciate differences among the three types of detection techniques (i.e., supervised, semi-supervised, and unsupervised), beside the A-ADL also N-ADL changing is considered; that is, to take into account the potential overlapping of more ADLs in the same sampling interval as well as the occurrence of ALDs never observed before.

### 3.2. Learning Techniques for Abnormal Behavior Detection

The problem of ABD can be addressed by means of several learning techniques. Fundamentally, the technique to be used depends on the label availability, so that it is possible to distinguish between the three main typologies of (1) super-vised detection; (2) semi-supervised detection; and (3) unsupervised detection, as is discussed in this subsection.

#### 3.2.1. Supervised Detection

**Figure 12:**
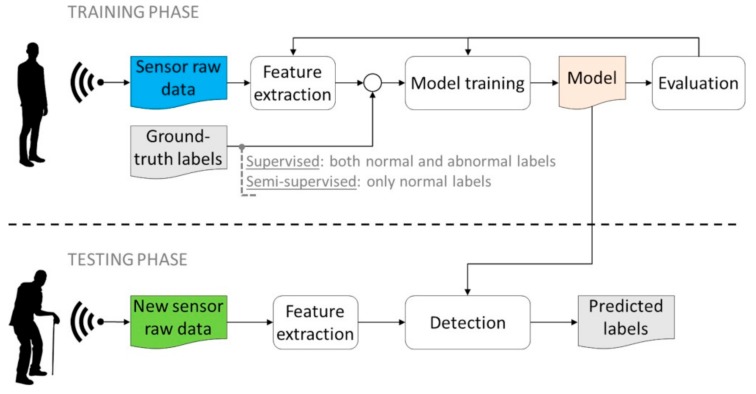
Workflow of supervised and semi-supervised detection methods. Both normal and abnormal labels are needed in the supervised training phase, whereas only normal labels are required in the semi-supervised training.

Supervised detection is based on learning techniques (i.e., classifiers) requiring fully labelled data for training. This means that both positive samples (i.e., ABs) and negative samples (i.e., normal behaviors) must be observed and labelled during the training phase. However, the two label classes are typically strongly unbalanced, since abnormal events are extremely rare in contrast to normal patterns that instead are abundant. As a consequence, not all classification techniques are equally effective for this situation. In practice, some algorithms are not able to deal with unbalanced data [30], whereas others are more suitable thanks to their high generalization capability, such as support vector machine (SVM) [31] and artificial neural networks (ANNs) [32], especially those with many layers like convolutional neural networks (CNNs), which have reached impressive performances in detection of AB from videos [33]. The workflow of supervised detection is pictorially depicted in Figure 12.

#### 3.2.2. Semi-Supervised Detection

In real-world applications, the supervised detection workflow described above is not very relevant due to the assumption of fully labelled data, on the basis of which abnormalities are known and labeled correctly. Instead, when dealing with elderly monitoring, abnormalities are not known in advance and cannot be purposely performed just to train detection algorithms (e.g., think, for instance, to falls in the elderly which involve environmental hazards in the home). Semi-supervised detection also uses a similar workflow of that shown in Figure 12 based on training and test data, but training data only involve normal labels without the need to label abnormal patterns. Semi-supervised detection is usually achieved by introducing the concept of one-class classification, whose state-of-the-art implementations—as experimented in this study—are one-class SVM (OC-SVM) [21] and auto-encoders (AEs) [22] within ML and DL fields, respectively. DL techniques learn features in a hierarchical way: High-level features are derived from low-level ones by using layer-wise pre-training, in such a way structures of every higher level are represented in higher layers of the network. After pre-training, a semi-supervised training provides a fine-tuning adjustment of the network via gradient descent optimization. Thanks to that greedy layer-wise pre-training followed by semi-supervised fine-tuning [34], features can be automatically learned from large datasets containing only one-class label, associated with normal behavior patterns.

#### 3.2.3. Unsupervised Detection

**Figure 13:**
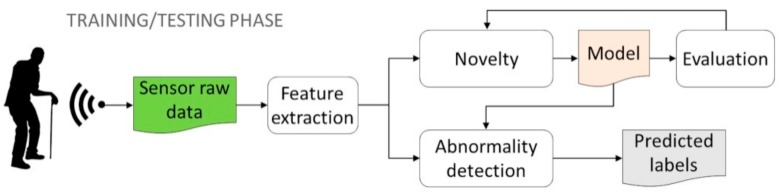
Workflow of unsupervised detection methods.

The most flexible workflow is that of unsupervised detection. It does not require that abnormalities are known in advance but, conversely, they can occur during the testing phase and are modelled as novelties with respect to normal (usual) observations. Then, there is no distinction between training and testing phases, as shown in Figure 13. The main idea here is that extracted patterns (i.e., features) are scored solely on the basis of their intrinsic properties. Basically, in order to decide what is normal and is not, unsupervised detection is based on appropriate metrics of either distance or density.

Clustering techniques can be applied in unsupervised detection. In particular, K-means is one of the simpler unsupervised algorithms that address the clustering problem by grouping data based on their similar features into K disjoint clusters. However, K-means is affected by some shortcomings: (1) sensitivity to noise and outliers; (2) initial cluster centroids (seeds) are unknown (randomly selected); and (3) there is no criterion for determining the number of clusters. The Weighted K-Means [24], also adopted in this study, provides a viable way to approach clustering of noisy data. The last two problems are addressed by implementing the intelligent K-means suggested by [25], in which the K-means algorithm is initialized by using the so-called anomalous clusters, extracted before running the K-means itself.

### 3.3. Experimental Setting

For the experimental purpose, 31,500 datasets were generated, i.e., 1500 random instances for each of the 21 abnormalities, obtained by considering the abnormalities (St, Du, Di, Sw, Lo, Hr), shown in Figure 6, Figure 7, Figure 8, Figure 9, Figure 10 and Figure 11, together with all pairs of these abnormalities taken without repetitions. Each dataset represented a 1-year data collection, as a matrix (image) of 365 rows (days) and 288 columns (samples lasting 5 min each), for a total amount of 105,120 values (pixels) through 120 levels. The feature extraction process was carried out by considering a 50%-overlapping sliding window lasting 25 days, then leading to a feature space of dimension D = 7200.

Each dataset was divided into three parts: Upper (1st–90th days), middle (90th–180th days), and lower (180th–365th days) regions. The feature vectors (i.e., ACT-LOC-HRL sequences) belonging to the upper regions were negative samples (i.e., normal behavior), whereas those belonging to the lower regions were positive ones (i.e., AB). The middle regions were, instead, considered as prediction regions, characterized by gradual changes becoming progressively more accentuated. The aim is to classify the feature vectors belonging to the middle regions in order to predict the likelihood of a future change which will become increasingly relevant and stable from the 180th day onwards (lower region).

In both supervised and semi-supervised settings, regarding the SVM classifier, a radial basis function (RBF) kernel was used. The kernel scale was automatically selected using a grid search combined with cross-validation on randomly subsampled training data [35]. Regarding the CNN-based supervised detection, the network structure included eight layers: Four convolutional layers with a kernel size of 3 × 3, two subsampling layers, and two fully connected layers. Finally, the two output units represented, via binary logical regression, the probability of normal and abnormal pattern behaviors.

The stacked auto-encoder (SAE) network was structured in four hidden layers, and the sliding-window feature vectors were given as input to the first layer, which thus included 7200 units (i.e., corresponding to feature space dimension D). The second hidden layer was of 900 units, corresponding to a compression factor of 8 times. The following two hidden layers were of 180 and 60 units, respectively, with compression factors of 5 and 3 times. In supervised detection settings, the six abnormal datasets were joined in order to perform a 6-fold cross-validation scheme. In semi-supervised detection settings, instead, only normal data from the same dataset were used for training, while testing was carried out using data from day 90 onwards.

Regarding the convolutional auto-encoder (CAE) structure in the deep clustering (DC) approach, the encoder included three convolutional layers with a kernel size of five, five, and three, respectively, followed by a fully connected layer. The decoder structure was a mirror of the encoder one. All experiments were performed on an Intel i7 3.5 GHz workstation with 16GB DDR3 and equipped with GPU NVidia Titan X using Keras [36] with Theano [37] toolkit for DL approaches, and Matlab [38] for ML approaches.

## 4. Results

**Figure 14:**
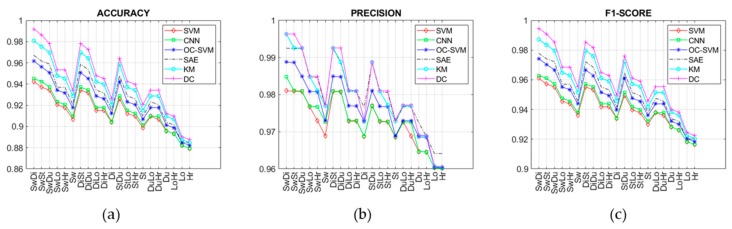
(**a**) Accuracy, (**b**) precision, and (**c**) F1-score of all compared techniques.

**Figure 15:**
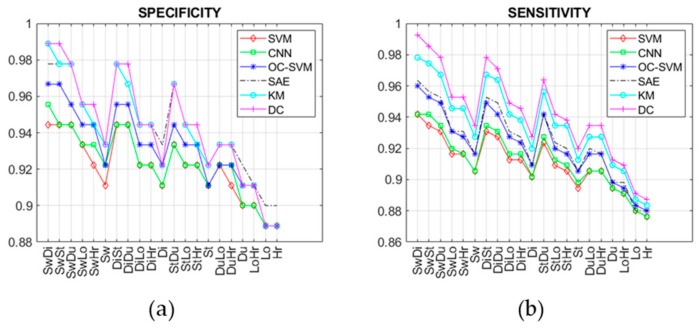
(**a**) Specificity and (**b**) sensitivity of all compared techniques.

**Figure 16:**
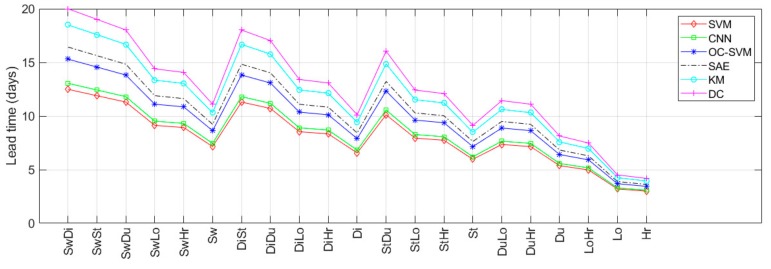
Lead-time of prediction of all compared techniques.

This section reports the experimental results in terms of detection accuracy, precision, sensitivity, specificity, F1-score, and lead-time of prediction related to all techniques summarized in Table 1, as well as the achieved processing of the datasets generated by considering 21 change types (i.e., SwDi, SwSt, SwDu, SwLo, SwHr, Sw, DiSt, DiDu, DiLo, DiHr, Di, StDu, StLo, StHr, St, DuLo, DuHr, Du, LoHr, Lo, Hr) as previously described. The achieved results are reported in Figure 14, Figure 15 and Figure 16, for each aforesaid performance metric and lead time. As discussed in the previous section, such abnormalities regard both N-ADLs and A-ADLs. The former regard the overlapping of different activities within the same sampling interval or the occurrence of new activities (i.e., sequences not observed before that may lead to misclassification). Instead, the latter take into account 21 types of combined changes from the usual activity sequence.

From Figure 14 and Figure 15, it is clear that with changes based on Lo, Hr, and Du there are little differences in detection performance, which become more marked with other kind of change such as Sw, Di, and St. In particular, the supervised techniques exhibit poor detection accuracy with change types as Lo and Hr, while the semi-supervised and unsupervised techniques based on DL maintain good performance also in correspondence of those change types. Similar considerations can be carried out by observing the other performance metrics (precision, F1-score, sensitivity, specificity). The change types Lo (Figure 10) and Hr (Figure 11) influence only a narrow region of the intensity values. More specifically, only location values (Figure 10b) are interested in Lo-type datasets, and only heartrate values (Figure 11b) in the Hr case. On the other hand, other change types like Di (Figure 8) or Sw (Figure 9) involve all values, i.e., ADL, LOC, and HRL, and so they are simpler to be detected and predicted.

The lead-times of prediction reported in Figure 16 were obtained in correspondence of the performance metrics discussed above and reported in Figure 14 and Figure 15. In other words, such times refer to the average number of days, before the 180th day (since from this day on, the new behavior becomes stable), at which the change can be detected with the performance reported in Figure 14 and Figure 15. The longer the lead-times of the prediction the earlier the change can be predicted. In this case, better lead-times were also achieved with abnormalities based on change types Di and Sw (i.e., characterized by wider regions of intensity variations) and with the SAE and deep clustering (DC) techniques, since they are able to learn discriminative features more effectively than the traditional ML techniques.

## 5. Discussion

Six different supervised, semi-supervised, and unsupervised learning techniques have been compared in the present study. In general, detecting ABs by using supervised methods presents the shortcoming of requiring both positive samples (i.e., changing activity sequences) and negative samples (i.e., habitual activity sequence) for model training. Positive samples, in particular, cannot be available when the detection system is used the first time for monitoring a new user. Therefore, supervised models need to be trained by using positive samples based on activities performed by different subjects, and thus characterized by a different probability distribution. This problem of lack of real data for training arises no matter what supervised learning techniques are used, i.e., classical ML or DL techniques. Typically, supervised techniques used in abnormality detection are SVM [18] and HMM [19], both falling into the non-parametric learning category. In this study, the SVM-based detection has been evaluated by training the supervised model with positive and negative samples taken from different datasets, in order to reproduce more accurately the real-life conditions mentioned above. Due to the lack of real data for training discussed above, the supervised detection approaches (SVM and CNN) achieved the lowest performances, as shown in Figure 14, Figure 15 and Figure 16. With semi-supervised techniques, the problem of training data availability is mitigated, since only negative samples (i.e., normal activity sequences) are required, which are quite abundant in everyday activities. In this case, the main difficulty is to select training samples that are most representative of normal behaviors. The semi-supervised approaches evaluated in this study, i.e., OC-SVM and SAE, achieved intermediary performances, as reported in Figure 14, Figure 15 and Figure 16, although with a significantly lower number of FP in the case of SAE. The most promising results were obtained with the unsupervised learning methods, i.e., K-means (KM) and DC, in which no labeled data were necessary, allowing the easy adaptability to different environmental conditions as well as to users’ physical characteristic and habits [23]. The unsupervised detection, however, required an initial calibration period, of about 60 days, during which the system was unable to detect changes from usual activity.

Classical ML methods, such as SVM and OC-SVM, have to deal with the problem of learning a probability distribution from a set of samples, which generally means to learn a probability density that maximize the likelihood on given data. However, such density does not always exist, as what happens when data lie on low-dimensional manifolds, which is the case of change types involving a narrow range of values (i.e., ADL, LOC, and HRL), or when training data come from a different probability distribution. Under such a point of view, conversely, DL methods are more effective because they follow an alternative approach. Instead of attempting to estimate a density, which may not exist, they define a parametric function (representing some kind of deep neural network (DNN)) able to generate samples. Thus, by (hyper-)parameter tuning, generated samples can be made closer to data samples taken from the original data distribution.

Instead, by exploiting the ability of deep neural networks (DNNs) to generate samples more and more close to the original data, through careful (hyper-)parameter tuning, the well-known “Big” data dimensions of volume, variety and velocity can be effectively exploited to improve detections [39]. In fact, the usage of massive amounts of data (volume) is one of the greater advantages of DNNs, which can be also adapted to deal with data abstraction in various different formats (variety) coming from sensors spread around a smart home environment. Moreover, clusters of graphic processing unit (GPU) servers can be used for massive data processing, even in real-time (velocity). However, the application of DL techniques for the purpose of anomaly (i.e., AB) detection is still in its infancy [40]. CNN, which is the current state-of-the-art in object recognition from images [20], exhibits very high feature learning performance but it falls into the category of supervised techniques. A more interesting DL technique for abnormal activity recognition is represented by AEs, and in particular the stacked AEs (SAEs) [22], which can be subsumed in the semi-supervised techniques when only normal samples are used for training. Furthermore, SAEs are basically unsupervised feature learning networks, and thus they can also be exploited for unsupervised anomaly detection. The main limitation of AEs is its requirement of 1D input data, making them essentially unable to capture 2D structures in images. This issue is overcome by the convolutional auto-encoder (CAE) architecture [41], which combines the advantages of CNNs and AEs, besides being suitable for DC tasks [42] and, thus, making it a valuable technique for unsupervised abnormal behavior detection (ABD).

In general, the worst accuracies were found in presence of change types Hr and Lo, due to the fact that such changes involve a narrow region of the intensity values. On the other hand, the detection accuracy improved in the presence of two kinds of changes, as one can easily see from Figure 14a. However, the ability of DL techniques to capture spatiotemporal local features (i.e., relations between activities) allowed good performance to be achieved also with change types whose intensity variations were confined in narrow regions.

## 6. Conclusions

The contribution of this study is twofold. First, a common data model able to represent and process simultaneously ADLs, home locations in which such ADLs take place (LOCs), and PPs (HRLs) as image data is presented. Second, the performance of state-of-the-art ML-based and DL-based detection techniques were evaluated by considering big data sets, synthetically generated, including both normal and ABs. The achieved results are promising and show the superiority of DL-based techniques in dealing with big data characterized by different kinds of data distribution. Future and ongoing activities are focused on the evaluation of prescriptive capabilities of big data analytics aiming to optimize time and resources involved in elderly monitoring applications.

## Figures and Tables

**Table 1:** Machine learning (ML) and deep learning (DL) techniques compared in this study.

Category	Type	Technique
Supervised	Machine learning	Support vector machine (SVM)
Supervised	Deep learning	Convolutional neural network (CNN)
Semi-supervised	Machine learning	One-class support vector machine (OC-SVM)
Semi-supervised	Deep learning	Stacked auto-encoders (SAE)
Unsupervised	Machine learning	K-means clustering (KM)
Unsupervised	Deep learning	Deep clustering (DC)

**Table 2:** Activities, home locations, and heartrate values used to generate the long-term datasets.

Activity of Daily Living (ADL)	Home Location (LOC)	Heartrate Level (HRL)
Eating (AE) Housekeeping (AH) Physical exercise (AP) Resting (AR) Sleeping (AS) Toileting (AT)	Bedroom (BR) Kitchen (KI) Living room (LR) Toilet (TO)	Very low (VL) [<50 beats/min] Low (LO) [65–80 beats/min] Medium (ME) [80–95 beats/min] High (HI) [95–110 beats/min] Very high (VH) [>110 beats/min]
